# Meeting the Needs of Different Research Communities

**DOI:** 10.1371/journal.pmed.0020158

**Published:** 2005-05-31

**Authors:** The PLoS Medicine Editors

## Abstract

The Public Library of Science is launching three new journals in 2005, led by academics and aimed at the needs of different research communities.

## Teaser

PLoS launches three new community journals in 2005

PLoS was founded in October 2000 not as a publisher, but as a grassroots movement of researchers who believed that the results of the global research enterprise should be a freely available public resource. So why did PLoS become a publisher, and what are we doing in this role to serve the world's varying biomedical researchers and clinicians?

At its inception, the first action by PLoS was to encourage scientific and medical journal publishers to make the archival research literature freely available. An open letter, signed by almost 34,000 scientists from 180 countries, urged publishers to deposit copies of their research articles in a full text public repository, such as PubMed Central, within six months of publication. Sadly, the vast majority of publishers declined to deposit their works—a depressing situation that continues to this day.

We concluded that the only way forward was to publish our own journals. These would provide an alternative, open-access venue for important discoveries in science and medicine and would serve as a model for showing that open-access publication is viable.

The first phase of our life as a publisher involved launching our two flagship journals—*PLoS Biology* in October 2003 and *PLoS Medicine* in October 2004. These two journals provide an open-access alternative to the best subscription journals in the life sciences and medicine, respectively.

These first journals have helped to put open access on the map. In the 18 months since we started as a publisher not only have many researchers embraced these fledgling journals as a top tier “home” for their work, but also there has been growing international support for open-access initiatives.

For example, 55 institutions worldwide have so far signed the Berlin Declaration on Open Access to Knowledge in the Science and Humanities. Beginning this month, researchers who are funded by the United States National Institutes of Health are being asked by the agency to deposit a copy of their accepted research papers into PubMed Central. And in the United Kingdom, the Wellcome Trust is making it a requirement of its grant conditions that Wellcome Trust–funded researchers deposit an electronic version of their manuscripts in a UK portal of PubMed Central within six months of publication.

PLoS is now entering its second phase as a publisher, in which we launch the first three PLoS community journals. The case for launching these journals was compelling—we wanted to serve three research communities that had few open-access alternatives to the subscription journals in their field. As a result, we are launching *PLoS Computational Biology* (www.ploscompbiol.org), a collaboration between PLoS and the International Society for Computational Biology, scheduled to start publishing in June 2005; *PLoS Genetics* (www.plosgenetics.org), scheduled for July 2005; and *PLoS Pathogens* (www.plospathogens.org), scheduled for September 2005.

With the arrival of the community journals, we are providing a greater range of open-access venues for researchers who wish to ensure that anyone can read, use, and build on their work. Unlike *PLoS Biology* and *PLoS Medicine*, which are run by PLoS editorial staff, each community journal is run by the community itself—that is, by an academic editor-in-chief and editorial board, with production support from PLoS staff. The community-led nature of the new journals, coupled with a business model in which publication costs are borne largely by publication charges, provides an example for other journals that wish to transition to open access.

When submitting their work to PLoS, how do researchers and clinicians distinguish between *PLoS Medicine* and the PLoS community journals? *PLoS Medicine* remains committed to publishing the best medical research that is relevant to a broad international community of clinicians and researchers. The community journals are aimed at a more specialized audience. *PLoS Pathogens*, for example, will publish basic scientific research that “significantly advances the understanding of pathogens and how they interact with their host organisms.” But we also think that *PLoS Medicine* readers will find much of interest in the community journals, and vice versa, and we will cross-link between articles in the different journals.

The different PLoS journals will be editorially independent and submissions will remain confidential to each journal. But if an author would like a manuscript that is not thought to be appropriate by one journal to be passed on to another, along with the reviewers' reports and their identities, we are happy to cooperate, subject to the permission of the reviewers. This can help to speed up the review process.

As we roll out the new community journals, we are already planning the third phase of our life as a publisher—the creation of an online repository for all technically sound research reports in both biology and medicine, including clinical trial reports. It is an ambitious plan, but one that we believe will provide authors with more choices and in the end, an open-access venue for the widest range of research.

